# Isolated low grade prenatally detected unilateral hydronephrosis: do we need long term follow-up?

**DOI:** 10.1590/S1677-5538.IBJU.2017.0474

**Published:** 2018

**Authors:** Osama M. Sarhan, Ahmed El Helaly, Abdulhakim Al Otay, Mustafa Al Ghanbar, Ziad Nakshabandi

**Affiliations:** 1 Urology and Nephrology Center, Faculty of Medicine, Mansoura University, Mansoura, Egypt; 2 Division Urology Pediatric, Prince Sultan Military Medical City, Riyadh, Saudi Arabia; 3 Mansoura Health Insurance Hospital, Mansoura, Egypt

**Keywords:** Hydronephrosis, Follow-Up Studies, Urinary Tract

## Abstract

**Purpose:**

To assess the need for postnatal evaluation and the medium term outcome in patients with isolated unilateral low grade prenatally detected hydronephrosis.

**Materials and Methods:**

We prospectively selected 424 patients (690 kidney units) with a prenatal diagnosis of urinary tract dilatation between 2010 and 2013. We included only those patients with isolated unilateral low-grade hydronephrosis who underwent at least 2 postnatal ultrasound examinations. The Society for Fetal Urology (SFU) grading system was utilized for assessment of the hydronephrosis. We excluded patients with bilateral dilation or other urological abnormalities. The fate of hydronephrosis including resolution, stability or worsening was documented.

**Results:**

A total of 66 infants (44 boys and 22 girls) with antenatally diagnosed unilateral urinary tract dilation (23 right and 43 left) were identified. Ultrasounds showed SFU grade 1 hydronephrosis in 32 patients (48%) and SFU grade 2 hydronephrosis in 34 (52%). After a mean follow-up period of 32 months (range 12 to 60), 37 patients (56%) had complete resolution of hydronephrosis while the remaining 29 were stable (44%). None of our patients developed UTIs during follow-up and none required surgical intervention.

**Conclusions:**

Prenatally detected, isolated unilateral low-grade hydronephrosis usually have a favorable prognosis. All cases in our cohort showed either stability or resolution of hydronephrosis without any harmful consequences. Based on our findings on medium-term in this category of patients, long-term follow-up is not warranted.

## INTRODUCTION

Before the era of antenatal screening and the use of prenatal ultrasound, most of the urologic anomalies were diagnosed only when they were symptomatic or complicated. Even to the extent that some patients might incidentally present with symptoms of end stage renal disease as their initial presentation (1). With the advancement of antenatal care, prenatal ultrasonography started to detect significant fetal anomalies during pregnancies. Out of these, 20-30% were attributed to urogenital anomalies and 50% were due to hydronephrosis (HN) (1, 2).

Hydronephrosis occurs in 1-5% of all pregnancies (3). Once diagnosed, the debate will arise whether it is obstructive or non-obstructive in nature, harmful to the kidney and whether any prenatal or postnatal surgical intervention will be required. Different etiologies have been attributed to the diagnosis of antenatal hydronephrosis (ANH) which might include, transient phenomena, pelvic ureteric junction obstruction, vesicoureteral reflux (VUR), posterior urethral valves or other anomalies (4).

The exact definition of significant ANH is evolving. Multiple grading systems and parameters were used to define this entity. However, the society for fetal urology (SFU) grading system and the measurement of the antero-posterior diameter (APD) of the renal pelvis were among the commonest utilized (5). Isolated unilateral low-grade HN is usually benign in nature and about 50% of fetuses and infants diagnosed with this entity might show complete resolution on follow-up (6). On the contrary, high-grade HN will usually require more extensive evaluation and strict follow-up (7). This might require the use of continuous antibiotic prophylaxis (CAP), further evaluation, longer follow-up period and they might require surgical intervention, depending on the underlying etiology (6–8).

In our study, we prospectively followed up infants diagnosed with ANH and confirmed postnatally to have isolated unilateral, low-grade (SFU grade 1 or 2) HN, in order to determine the necessity for further evaluation, follow-up and outcome.

## MATERIALS AND METHODS

After obtaining the approval of institutional review board at Prince Sultan Military Medical City, a prospective study design was constructed. Signed parental consent for participation was obtained before the start of the study. Study inclusion period started from January 2010 to December 2013.

A total of 522 fetuses diagnosed with antenatal AHN were selected for postnatal screening. Infants were referred prospectively after routine detection in the maternity unit with a mean gestational age at diagnosis of 33 weeks (range 28 to 36 weeks). All of these infants had complete physical examination (to exclude any other significant congenital abnormalities) in addition to a postnatal ultrasound (US) performed after the first week and within the first month of life. This first performed postnatal US was used as a baseline for assessing the presence of HN and only including those with unilateral low-grade HN and excluding any patient with other suspected anomalies, namely: duplication anomaly, multicystic dysplastic kidney, dilated ureter, ureterocele, ectopic ureter, urinary bladder diverticulum, thickened bladder wall or posterior urethral valves. The SFU grading system was used to classify all patients independently by at least two physicians, a pediatric radiologist, and a pediatric urologist, to minimize inter-reviewer variability. If physicians disagreed, the higher grade was taken.

Of the 522 patients (788 renal units) with prenatal HN, 98 patients were excluded due to two consecutive normal postnatal renal US (absent HN). Additional 275 patients (550 renal units) were excluded due to bilaterality. Of the 149 patients with unilateral HN, 83 cases were excluded, 44 with high-grade HN and 39 were found to be associated with other urological anomalies postnatally. Our study included 66 patients, with isolated unilateral low-grade HN (SFU grade 1 and SFU grade 2) who met our study criteria.

**Figure 1:**
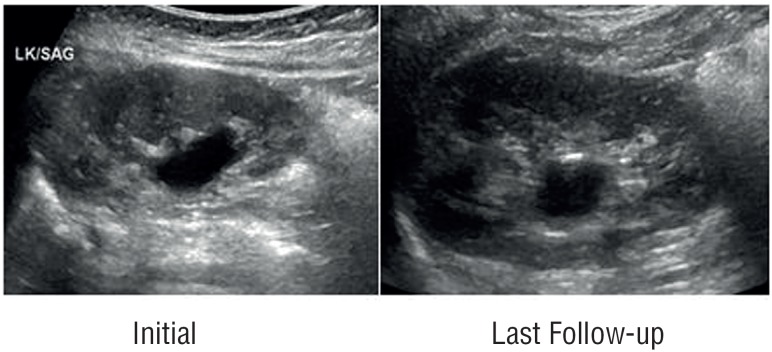
Renal US image in a patient with initial grade-1 HN and last follow-up US showing persistent grade-1 HN.

**Figure 2:**
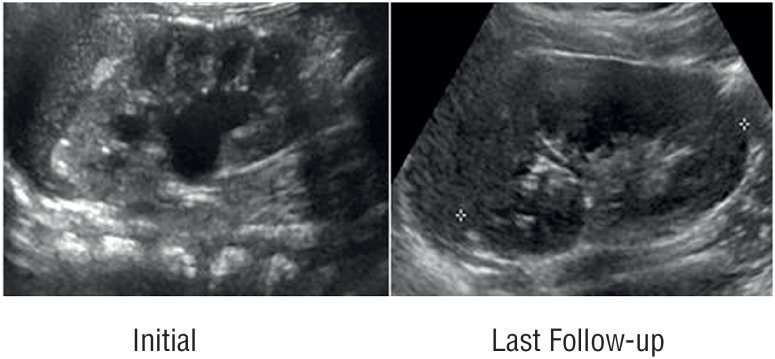
Renal US image in a patient with initial grade-2 HN and last follow-up US showing resolution of HN.

Further follow-up plan for our cohort of patients incorporated performance of US at 6, 12 months and annually thereafter. Patients were kept off any CAP and were monitored for growth parameters and occurrence of any signs and symptoms of febrile urinary tract infections (UTI's) by their care givers and pediatricians. Urine cultures were performed if there were any suspected symptoms of UTI's. If any febrile urinary tract infection was documented, it was recorded and additional screening for vesicoureteral reflux by voiding cystourethrogram (VCUG) was planned. Further follow-up US were utilized to identify patients who had a complete resolution, remained stable or had progression of their HN. We identified resolution as complete regression of HN, stability as same or downgraded HN, progression as upgraded HN. Figure 1 and 2 showed both initial and last follow-up renal US in two children with grade 1 and 2, respectively.

Statistical analysis was performed using SPSS version 21 (SPSS Inc., Chicago, IL, USA). We used binary and logistic regression analysis. Values are shown as mean±SD unless otherwise reported. Kaplan-Meier estimates were used to show the effect of HN grade, side, and gender on the resolution rate of HN and compared with the use of the log-rank test. All tests were 2-sided. A p value of less than 0.05 was considered statistically significant.

## RESULTS

All of our 66 patients with isolated unilateral low-grade HN completed the study. The mean follow-up period was 32 months (range, 12-60). Patient's demographics are shown in Table-1.

**Table 1:** Patients’ Demographics.

Unilateral isolated low grade HN		Number No. = 66	Percentage %
**Gender**			
	Male	44	67
	Female	22	33
**Laterality**			
	Right	23	35
	Left	43	65
**SFU grade**			
	Grade 1	32	48
	Grade 2	34	52

**HN** = Hydronephrosis; **SFU** = Society for Fetal Urology

**Figure 3:**
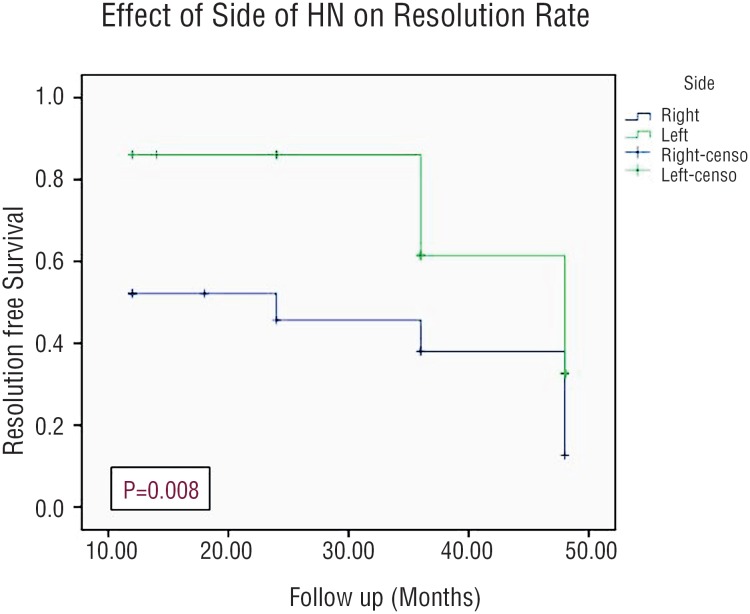
Kaplan-Meier Curve Showing Incidence of Resolution Stratifying By Side of HN.

**Figure 4:**
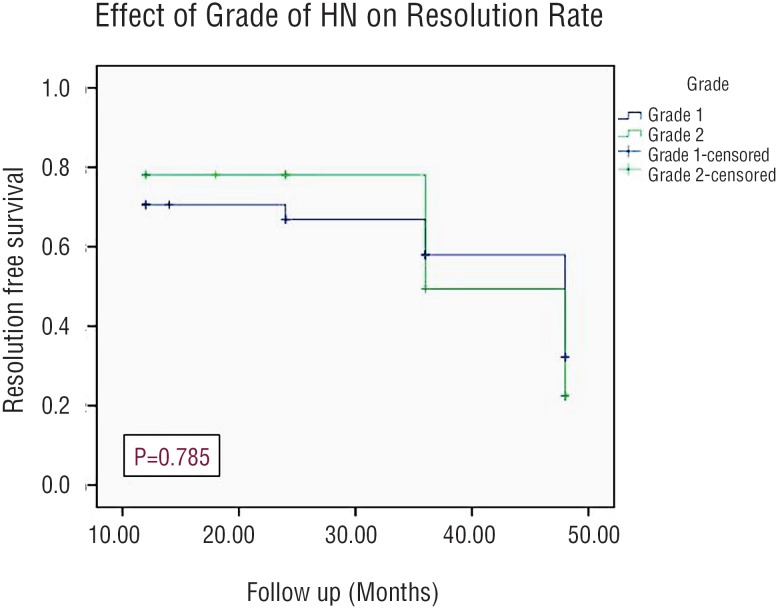
Kaplan-Meier curve Showing Incidence of Resolution Stratifying By Grade of HN.

**Figure 5:**
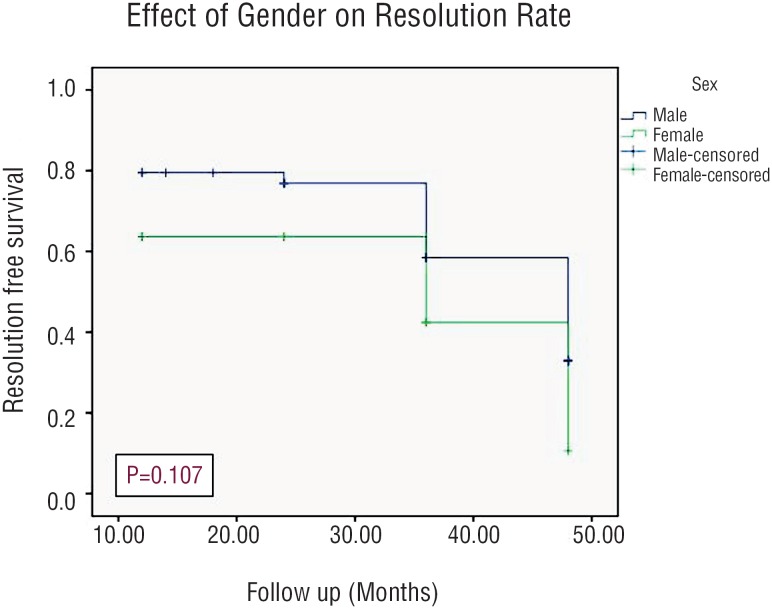
Kaplan-Meier curve Showing Incidence of Resolution Stratifying By Gender.

Resolution rate was 56% with a mean time for resolution was 13.5 months (range, 6-36). Using Kaplan-Meier estimates, the side of HN had a statistically significant effect on the resolution rate of HN (p=0.008), while the grade of HN and patients gender did not have any statistically significant impact on the resolution rate (p=0.785 and 0.107, respectively) as shown in Figures 3–5.

During the course of follow-up, none of our patients had relapse/progression of their HN, developed UTI's (either febrile or afebrile), or required another mode of investigation. Additionally, none required any form of surgical intervention.

## DISCUSSION

With the improvement in health care facilities and ultrasound technology, fetal HN could be detected between 12^th^ to 14^th^ weeks of gestation and now readily diagnosed by maternal antenatal screening. The rationale for following patients with antenatal AHN is to avoid hazardous drawbacks on the patient's kidneys. Many controversies coexist on how best to manage antenatal HN, which arise from the incomplete understanding of the natural history of this anomaly (5). In the past, evaluation included postnatal US, VCUG and often renal scans, so that there was an increase in the yield of abnormalities that is deemed to be clinically insignificant (8). Other controversies were the debate on the usage of CAP with the lack of evidence on benefits over a prolonged follow-up interval (9).

In our study, we aimed to identify all patients with a history of antenatal AHN, evaluate them postnatally, and then prospectively follow those with isolated unilateral low-grade HN to determine the fate and proposed follow-up plan for them. We used the first postnatal US as the base line for categorization of these patients since we believe, as many others do, that in experienced hands, the first detailed postnatal US is the corner stone imaging to establish the grade of HN and to decide on further investigation (10). Other studies used the prenatal APD of the renal pelvis as the base for categorization of patients postnatally and determining further work-up and follow-up plans (11).

Of the 522 fetuses diagnosed with antenatal AHN in our study, 98 (18.7%) were found to have a normal first postnatal renal US as well as a second confirmatory scan within the first year of life and hence were excluded from our study. This group of infants has a very low incidence of complications as reported by many studies (12, 13). In a study on 143 infants with antenatal AHN, 50 of them had normal postnatal sonograms without HN. Only three were later diagnosed with VUR (all were low grade) (12). Similarly, in another study including 103 infants with ANH, 53 infants had a normal postnatal renal US and only 3 had VUR (grade-I). The conclusion of both studies indicated that VUR was infrequent in this subset of infants and carries a good prognosis (13).

The need for starting CAP and performing VCUG in patients with isolated unilateral low-grade HN is a debatable issue. Some authors recommend starting CAP and performing VCUG in all neonates with antenatal ANH (14–16), while others do not recommend this approach (17, 18). The American Academy of Pediatrics (AAP) and the National Institute for Health and Care Excellence (NICE) guidelines as well as the Italian society of pediatric nephrology excluded the routine use of antimicrobial prophylaxis for low-grade VUR, concluding that in mild and moderate isolated hydronephrosis the search for VUR becomes clinically irrelevant (19, 20). Based on these data, we did not start any of our patients with CAP nor did we perform routine VCUG.

Out of the 66 patients in our study, resolution of HN was noticed in 37 patients (56%) with a mean time to resolution of 13.5 months and this rate of resolution coincided with those reported in several recent studies (6, 21, 22). In addition, there was no significant difference between the grades of HN and the gender of patients regarding the rate of resolution. However, resolution rate in relation to the side was statistically significant, which might be attributed to a statistical error related to proportionally small sample size.

Gökaslan et al. (2012), in their prospective study which included 49 patients with (SFU grade 1 and 2), with a follow-up period of 18 months, 64% showed spontaneous resolution. They strongly suggested that low-grade HN is a relatively self-limited condition and needs minimal investigations (21). Furthermore, Madden-Fuentes and associates (2014) published in their retrospective review of 623 kidney units, with a mean follow-up 14.6 months, an overall resolution rate (SFU grade 1 and 2) of 60% with no significant difference between grade 1 and 2 (6). They concluded that low-grade HN diagnosed within the first year of life remained stable or improved in 97.4% of renal units. They recommended observation for 12 months after diagnosis unless HN completely resolves (6).

In another prospective study performed by Coelho and colleagues in 2007, 192 patients were included, 89 with low-grade HN and were followed for a median period of 24 months. They found that cumulative rate of resolution of mild HN was 60% after 5 years follow-up (7). Similarly, Tombesi and Alconcher (2012), reported their results on 227 renal units with mild prenatally diagnosed HN. They found that 73% of their cohort showed complete resolution, 26% continued to be stable and only 1% deteriorated (22). Also, Barbosa and colleagues reported on 144 patients with mild HN and found 46.5% of them had a complete resolution in comparison to 47.2% who remained stable (23). In another meta-analysis by Sidhu et al., they reported that 98% of all mild hydronephrosis resolved, stabilized, or improved on follow-up, emphasizing favorable outcome of this patient category (24).

In a study with long-term follow-up (142 months) carried on isolated antenatal HN by Yang et al. (2010), with a median follow-up 142 months, results showed stabilization in all children with grade 1 HN and in 87% of children with grade 2 HN. The mean interval to spontaneous resolution was 13.4 months. They suggested that there is no need for invasive procedures and recommended observing patients closely during the first 2 years of life (25). On the other hand; none of our patients had relapse/progression of their HN or developed UTI's during the follow-up period. In contrast to our study, several investigators reported progression rate between 1-13% and that may be due to longer follow-up period and different inclusion criteria in their studies (6, 7, 20–25).

Though we have chosen not to start any patients on continuous antibiotic prophylaxis (CAP) based on the low rate of UTI in cases with low-grade HN, none of our patients developed UTI's (26). Madden-Fuentes et al., reported a UTI rate of 8.9% with isolated unilateral low-grade HN (6). Coelho et al. reported a UTI rate of 7.8% despite CAP (7). Others reported an incidence of 1.3 to 12% (27, 28). Given the low rate of UTI in their subset of patients, they concluded that antibiotic prophylaxis has a limited role in low-grade HN management (6). Moreover, all boys included in our study had ritual circumcision early in their life that might have an impact on the risk of UTI incidence in our cohort.

We had some limitations in our study including a relatively small number of patients with highly selective inclusion criteria. Selections were based on a subjective SFU grading system (as a single parameter) in addition to the inherent limitations of a non-randomized, non-blinded study without a control group. Measurements of biomarkers for obstruction were not used. UTI may be underestimated as it was evaluated by primary health care provider based on clinical symptomatology only.

## CONCLUSIONS

Isolated unilateral low-grade HN is a benign entity with no risk of significant morbidity. Patients of this category usually will not require invasive investigations or CAP. We recommend performing at least 2 postnatal US within the first year of life and no further evaluation if they showed resolution or stability of HN. Long-term follow-up is not warranted in the majority of cases and family reassurance will be sufficient.

## ABBREVIATIONS

ANH: Antenatal Hydronephrosis
CAP: Continuous Antibiotic Prophylaxis
HN: Hydronephrosis
SFU: Society for Fetal Urology
US: Ultrasound
UTI's: Urinary Tract Infections
VCUG: Voiding Cystourethrogram
VUR: Vesicoureteral Reflux
